# Clinico-radiological Analysis and outcomes of management of pineal region space occupying lesions: A multi-departmental, retrospective case series from Pakistan

**DOI:** 10.12669/pjms.41.13(PINS-NNOS).13378

**Published:** 2025-12

**Authors:** Haseeb Mehmood Qadri, Arooj Kiran, Raahim Bashir, Talha Sajid, Zia ul Rehman Najeeb, Zubair Mustafa Khan, Usman Ahmad Kamboh, Rabia Saleem, Abdul Majid, Syed Shahzad Hussain Shah, Asif Bashir

**Affiliations:** 1 Dr. Haseeb Mehmood Qadri, MBBS. Punjab Institute of Neurosciences, Lahore, Pakistan; 2 Dr. Arooj Kiran, MBBS. Punjab Institute of Neurosciences, Lahore, Pakistan; 3 Dr. Raahim Bashir. MBBS. Punjab Institute of Neurosciences, Lahore, Pakistan; 4 Dr. Talha Sajid, MBBS. Punjab Institute of Neurosciences, Lahore, Pakistan; 5 Dr. Zia ul Rehman Najeeb, MBBS. Punjab Institute of Neurosciences, Lahore, Pakistan; 6 Dr. Zubair Mustafa Khan, MBBS, FCPS. Punjab Institute of Neurosciences, Lahore, Pakistan; 7 Dr. Usman Ahmad Kamboh, MBBS, FCPS. Punjab Institute of Neurosciences, Lahore, Pakistan; 8 Dr. Rabia Saleem, MBBS, FCPS. Punjab Institute of Neurosciences, Lahore, Pakistan; 9 Dr. Abdul Majid, MBBS, FCPS. Punjab Institute of Neurosciences, Lahore, Pakistan; 10.Dr. Syed Shahzad Hussain Shah, MBBS, FCPS. Punjab Institute of Neurosciences, Lahore, Pakistan; 11.Prof. Asif Bashir, MBBS, MD, FAANS, FACS. (Diplomat American Board of Neurosurgery) Punjab Institute of Neurosciences, Lahore, Pakistan

**Keywords:** Neurosurgical Procedures, Pakistan, Pineal tumors, Treatment outcome

## Abstract

**Background and objective:**

Pineal region tumors are rare intracranial lesions accounting for less than one percent, with heterogeneous histopathology. This study aimed to provide a comprehensive overview of pineal region lesions, their clinical and radiological presentation, and their management and outcomes.

**Methodology:**

This was a retrospective case series conducted at the Departments of Punjab Institute of Neurosciences. The study included all patients who underwent surgical excision or supportive procedures (cerebrospinal fluid [CSF] diversion and biopsy) for pineal region space-occupying lesions (SOLs) between January 1, 2022, and December 31, 2024. A non-probability consecutive sampling technique was used to enroll all the patients who fitted inclusion criteria, during the defined study period. A total of 32 patients were included in the final analysis.

**Results:**

A total of 32 patients were reviewed, 68.8% (22) males and 31.3%(10) females with a mean age of 24.1±12.3 years. The most common presentation was headache in 87.5% (28). Radiologically, the lesion was hypodense in 81.3% (26) on computed tomography (CT). On magnetic resonance imaging (MRI), 34.4% (11) were hypointense on the T1 weighted images and 90.6% (29) were hyperintense on the T2 weighted images, and 53.1%(17) was homogenously enhanced on contrast. Among all, 40.6% (13) had surgical excision,46.9% (15) had only biopsy, and 6.3%(2) underwent cerebrospinal fluid (CSF) diversion surgery. On histopathology, 21.9% (7) were pineocytoma, 21.9% (7) were germinomas,12.5% (4) were pineoblastomas. Post-operative weakness occurred in 6.2% (2) and cerebrospinal fluid (CSF) leak occurred in 3.1%(1) patients.

**Conclusion:**

This study reinforces the clinical and surgical complexity of managing pineal region tumors, which present with symptoms of increased intracranial pressure. Continued integration of minimally invasive techniques, neuronavigation, and multidisciplinary care is essential for optimizing outcomes in this diverse tumor group.

## INTRODUCTION

Pineal region tumors are rare intracranial tumors, accounting for 2.8% to 11% and 0.4% of all primary brain tumors in the pediatric and adult populations, respectively.^1^ Among these tumors, pineal parenchymal tumors and germ cell tumors (GCT) represent the most frequent types of lesions. In addition, pineal parenchymal tumors include five distinct histotypes, which include pineocytoma, pineal parenchymal tumors of intermediate differentiation, papillary tumor of the pineal region, pinealoblastoma, and desmoplastic myxoid tumor of the pineal region, SMARCB1-mutant.^2^,^3^ GCTs include germinoma, embryonal carcinoma, yolk sac tumor, choriocarcinoma, teratoma, mixed GCTs.^2^,^3^ These tumors show rapid growth and usually present as non-communicating hydrocephalus, headache, and cerebellar signs.^3^

Surgery remains the cornerstone of treatment, ranging from biopsy to gross total resection. Following surgery, further treatment depends on the histological subtypes, grading, and extent of disease, in combination with chemotherapy or radiotherapy, as radiotherapy remains an essential component of the multidisciplinary treatment approach for most pineal region tumors.^2^ In addition to treatment for localized germinoma, the current standard of care is chemotherapy followed by reduced-dose whole ventricular irradiation plus a boost to the primary tumor. For pinealoblastoma patients, postoperative radiation has been associated with higher overall survival. However, non-resectable pineal region tumors are associated with obstructive hydrocephalus, which requires surgical management via ventricular internal shunt or endoscopic third ventriculostomy.^4^,^5^

Despite the existing literature, gaps remain in understanding the comprehensive management and long-term outcomes of pineal tumors, particularly within the Pakistani context. This study aimed to address these gaps by providing the first detailed comprehensive analysis of clinic-radiological presentation and elaborating on the management of pineal region tumors at the highest-volume center of Pakistan. By doing so, it seeks to contribute valuable insights into the epidemiology and treatment of these challenging tumors, ultimately enhancing patient care and outcomes in this demographic.

## METHODOLOGY

This was a retrospective case series conducted at the departments of Punjab Institute of Neurosciences. The study included all patients who underwent surgical excision or supportive procedures (cerebrospinal fluid [CSF] diversion and biopsy) for pineal region space-occupying lesions (SOLs) between January 1, 2022, and December 31, 2024. A non-probability consecutive sampling technique was used to enroll all the patients who fit the inclusion criteria during the defined study period. A total of 32 patients were included in the final analysis.

### Ethical approval:

It was obtained from the Institutional Review Board of Punjab Institute of Neurosciences (reference no. 2071/IRB/Approval/2025, dated February 12, 2025).

### Inclusion Criteria

- All the patients who underwent surgical excision or supportive procedures (cerebrospinal fluid (CSF) diversion and biopsy) for pineal region SOLs.

### Exclusion Criteria

- Patients with deficient medical records and patients who were lost to follow-up before 12 weeks were excluded from the study.

### Operational Definitions

- The pineal region refers to the anatomical area centered on the pineal gland and immediately adjacent midline structures, including the posterior third ventricle, quadrigeminal cistern, and dorsal midbrain (tectal plate). Lesions arising from or extending into this region were included, such as those involving the pineal gland itself, tectal plate, posterior falx, and adjacent falcine attachments, provided they were contiguous with or directly impacted the pineal region on imaging
- Gross total resection was defined as the complete tumor removal with no residual on postoperative MRI, near total resection is removal of more than 90–95% of the tumor with only minimal residual on imaging, and subtotal resection is removal of less than 90% of the tumor with visible residual on imaging.
- Types of lesion on basis of location: diffuse: infiltrative, ill-defined margins, spreading into nearby brain tissue, and bilateral: affecting both sides, often symmetrical spread across the midline.

The data collection procedure involved reviewing patient medical records, radiological investigations, operative notes, and follow-up documentation available in the hospital system. Data was extracted using a structured questionnaire developed by the research team. The data collection instrument was based on physical patient files, digital hospital records, operative logs, and clinic follow-up registers. Google Forms (Google Inc., USA) were used to collect the data. Each patient was assigned a unique study ID, and data were anonymized and were accessible only to designated research personnel.

### Data analysis technique:

The data were analyzed using IBM SPSS Statistics. Categorical variables such as gender, type of surgical procedure, and histopathological diagnosis were expressed as frequencies and percentages. Continuous variables like age, pre-operative and post-operative Karnofsky Performance Score (KPS) were presented as means ± standard deviation (SD).

## RESULTS

In this study, we included 32 patients with pineal SOL, among whom 68.8% (22) were males and 31.3% (10) were females. The mean age of the included patients was 24.1±12.3 years. The most common presenting symptom was headache, found in 87.5% (28) of patients, followed by vomiting in 78.5% (25), and decreased vision was found in 56.3% (18). The pre-operative Glasgow coma scale (GCS) was between 13-15 in 100% (32) patients, with a Karnofsky performance status (KPS) of 90.6±11.6, (Table-I).

**Table-I:** Descriptive analysis of the demographics and clinical presentation of patients with pineal lesions.

Variables	Percentage with Frequencies
***Symptoms at Presentation, % (n)***	
Headache	87.5 (28)
Vomiting	78.1 (25)
Photophobia	28.1 (9)
Decreased vision	56.3 (18)
Impaired memory	12.5 (4)
Focal cranial nerve deficit	6.3 (2)
Focal Motor deficit	9.4 (3)
Loss of consciousness	9.4 (3)
Syncope	9.4 (3)
Fecal or urinary incontinence	6.3 (2)
Pre-operative KPS, mean ± SD	90.6±11.6

**Fig.1:**
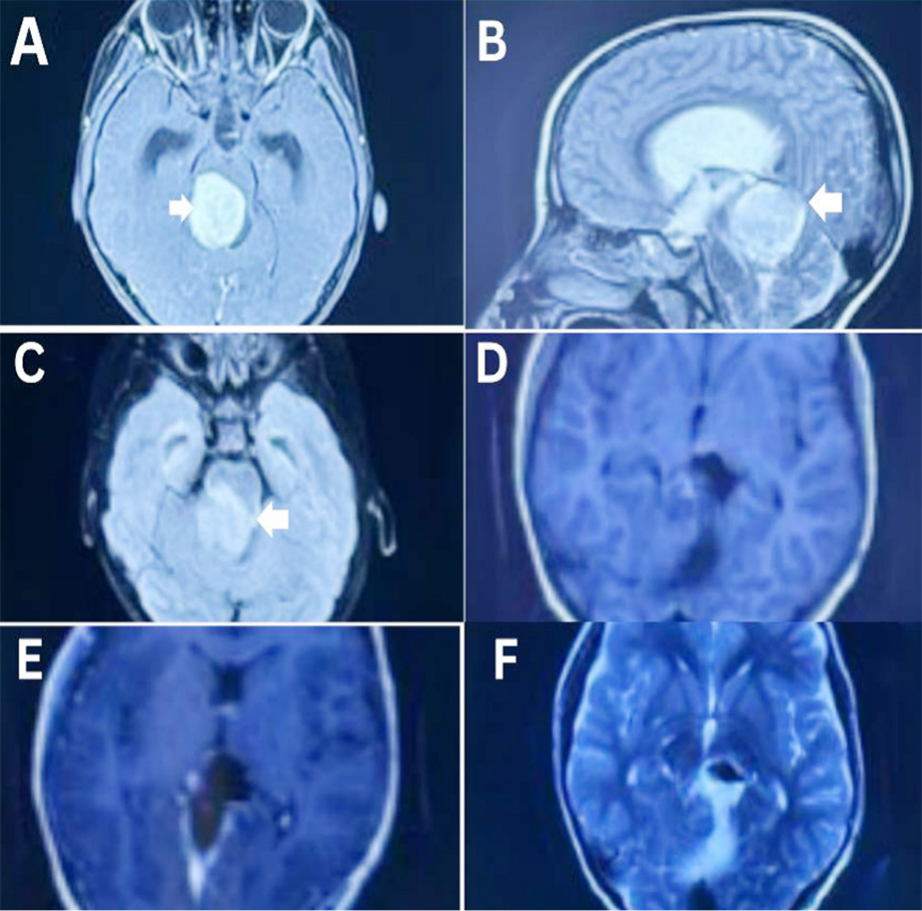
Pre-operative and post-operative MRI of 7-year-old male patient operated for Pineal sol with histopathology showing pilocytic astrocytoma, WHO grade 1. A: Axial T1WI Contrast pre-operative B: Sagittal T2WI preoperative C: Axial FLAIR pre-operative D: Axial T1WI post-operative E: Axial T1WI contrast post-operative F: Axial T2WI post-operative. White arrows are showing the lesion in A,B,C.

**Fig.2:**
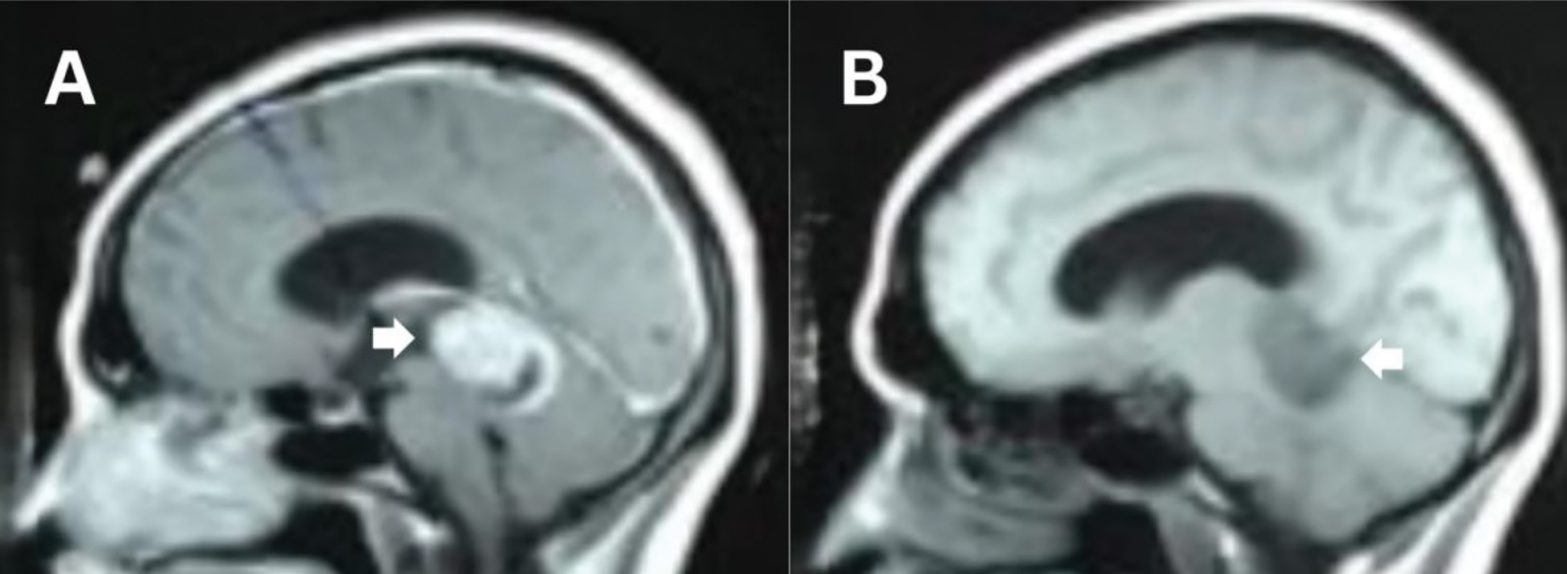
Pre-operative MRI of 30-year-old female patient operated for Pineal SOL showing histopathology of pineocytoma Grade1. A: Sagittal T1WI with contrast B: Sagittal T1WI plain. White arrows are showing the lesion.

The analysis of pre-operative imaging of the included patients showed that 90.6% (29) had a lesion in the pineal region, while 6.3% (2) had a lesion in the falx, and 3.1% (1) had a lesion in the tectum. The location of the lesion was midline in 90.6% (29); however, 6.3% (2) had bilateral lesions, and only 3.1% (1) had a diffuse type of lesion. On CT brain plain, 81.3% (26) of patients had hypodense lesions, while 15.6% (5) had hyperdense lesions, and only 3.1% (1) had isodense lesions. The T1-weighted images (T1WI) MRI showed 43.8% (14) hyperintense, 34.4% (11) hypointense, and 21.9% (7) isointense lesions; the T2-weighted images (T2WI) showed 90.6% (29) hyperintense, 6.3% (2) isointense, and 3.1% (1) hypointense lesions. About contrast, 53.1% (17) showed homogeneous uptake, 40.6 % (13) had heterogeneous uptake, and only 6.3% (2) had moderate uptake, (Table-II, Fig.1 and 2).

**Table-II:** Descriptive analysis of pre-operative neuroimaging of patients with pineal lesions.

***Site of SOL, % (n)***	
Pineal	90.6 (29)
Falx	6.3 (2)
Tectum	3.1 (1)
***Side of SOL, % (n)***	
Midline	90.6 (29)
Bilateral	6.3 (2)
Diffuse	3.1 (1)
***CT Findings, % (n)***	
Hypodense	81.3 (26)
Isodense	3.1 (1)
Hyperdense	15.6 (5)
***MRI T1 Findings, % (n)***	
Hypointense	34.4 (11)
Isointense	21.9 (7)
Hyperintense	43.8 (14)
***MRI T2 Findings, % (n)***	
Hypointense	3.1 (1)
Isointense Hyperintense	6.3 (2) 90.6 (29)
***MRI Contrast Findings, % (n)***	
Homogenous	53.1 (17)
Heterogeneous	40.6 (13)
Moderate	6.3 (2)

Analysis of the collected data revealed that 28.1 % (9) of patients needed a CSF diversion procedure preoperatively. Consistency of the excised tissue was soft in 90.6% (29), and firm in 9.4% (3) of the samples. Gross total resection was only possible in 9.4 % (3) patients, and near-total resection was achieved in 12.5% (4) patients; 3.1% (1) patients had subtotal resection, 62.5% (20) patients had partial resection, and in 12.5% (4) only biopsy was possible. Histopathology of the studied tissues showed that 21.9% (7) were pineocytomas, 12.5% (4) were pineoblastoma, 21.9% (7) were germinoma, and 9.4% (3) were meningioma. For 12.5 % (4) of patients, histopathology was not available. Immediate post-operative GCS was 13-15 in 93.7% (30) of patients and 9-12 in 6.3% (2). Immediate post-operative powers were 5/5 in all four limbs in 93.7% (30) and 4/5 in right upper and lower limbs in 6.3% (2) same as pre-operative. Post-operative complications included CSF leak in 3.1 % (1) and hydrocephalus in 21.9% (7) patients. Post-operative KPS was 91.5±15.0, (Table-III).

**Table-III:** Descriptive analysis of parameters of surgical management and post-operative sequelae of patients with pineal lesions.

Need for pre-operative CSF diversion? % (n)	28.1 (9)
Excisional Surgery, % (n)	40.6 (13)
Diagnostic / Biopsy Surgery, % (n)	46.9 (15)
***Non-excision / CSF diversion surgery, % (n)***	
VP shunt	6.3 (2)
EVD	37.5 (12)
***Consistency, % (n)***	
Soft	90.6 (29)
Firm	9.4 (3)
Hard	0 (0)
***Extent of Resection, % (n)***	
GTR	9.4 (3)
NTR	12.5 (4)
STR	3.1 (1)
PR	62.5 (20)
Biopsy	12.5 (4)
***Histopathology, % (n)***	
Pineocytoma	21.9 (7)
Pineoblastoma	12.5 (4)
Germinoma	21.9 (7)
Meningioma	9.4 (3)
Non-germinomatous germ cell tumor	6.3 (2)
Papillary tumor	6.3 (2)
Teratoma	3.1 (1)
Ependymoma	3.1 (1)
N/A	12.5 (4)
***Post-operative GCS, % (n)***	
3-8	0 (0)
9-12	6.3 (2)
13-15	93.7 (30)
***Post-operative Neurology, % (n)***	
5/5 in all limbs	93.7 (30)
4/5 on right side	6.3 (2)
***Post-operative Complications, % (n)***	
New Neurologic Deficit	0 (0)
Surgical Site Infection	0(0)
CSF Leak	3.1 (1)
Hydrocephalus	21.9 (7)
Others	0 (0)
Post-operative KPS, mean ± SD	91.5±15.0

GTR: gross total excision, NTR: near total excision, STR: subtotal excision, PR: partial excision, KPS: Karnofsky Performance Status.

## DISCUSSION

Pineal region tumors (PRT) encompass a wide variety of pathological types and exhibit unique epidemiological features. These are generally tumors of the pediatric and young adult populations at a peak incidence of 0-19 years. Our cohort also shows that the mean age of diagnosis was 24.1 ± 12.3 years.^6^ In contrast, PRT account for less than 1% of all adult intracranial tumors, of which pineal parenchymal tumors and gliomas are the most frequently encountered neoplasms in adults over the age of 40.^2^ Bin Alamer O et al. found the median age of pineal gliomas to be 39 years from a systematic review of 81 patients.^7^ Conversely, germ cell tumors (GCT)– the most common of which are germinomas, which also represent the most prevalent PRT altogether– are seen more often in children, comprising 11.8% of all pediatric primary intracranial tumors.^8^-^10^ Consistent with our findings of a 68.8%(22) male demographic, the existing literature shows that most PRT–especially GCT– demonstrate a male predilection ranging up to 70-94%.^6^,^9^-^12^,

The clinical presentation of PRT is largely a result of its mass effect with resultant obstructive hydrocephalus. In our cohort, headache 87.5%(28) and vomiting 78.1%(25) were the most common presenting symptoms, consistent with the broader literature where these manifestations, along with visual disturbances and signs of Parinaud syndrome, are frequently observed.^13^,^14^ Radiologically, 90.6%(29) of lesions were midline and primarily involved the pineal region, while CT and MRI demonstrated a predominance of hypodense and the T1/T2 hyperintense characteristics, respectively. Such imaging features can offer clues to tumor grade and histology; for instance, hyperintensity on the T2WI and heterogeneous contrast uptake may reflect higher grade tumors, while diffusion restriction on diffusion weighted image and apparent diffusion coefficient (DWI/ADC) shows higher cellularity and is typical of more aggressive tumors.^15^,^16^ Although tumor size was not explicitly measured, previous studies associate larger tumors (≥3 cm) with higher-grade pathology as well as infiltration into adjacent structures, which is a feature of some gliomas and pineal parenchymal tumor of intermediate differentiation (PPTID).^7^,^11^,^17^ The presence of hydrocephalus can be up to 60-92% in patients with PRT–usually of the non-communicating type due to compression of the cerebral aqueduct, and often necessitates pre-operative endoscopic third ventriculostomy (ETV) or Ventriculoperitoneal shunt (VPS).^7^,^18^

The surgical approach to PRT must balance optimal exposure with preservation of deep venous structures. While our study did not explicitly document the operative corridor used, the supracerebellar infratentorial (SCIT) approach remains the most commonly utilized for midline pineal lesions, offering a direct path to the pineal region with minimal cortical insult.^19^,^20^ The occipital transtentorial (OT) route may be preferred in cases with significant superior or posterior extension. Modern positioning techniques, such as park-bench with head elevation, are increasingly employed in endoscopic SCIT to enhance operative angles.^20^ Craniotomy size and trajectory vary by tumor location and chosen approach; recent literature reflects a shift from wide, traditional exposures to more targeted, keyhole-style craniotomies, such as the 3×3 cm bone window described in endoscopic SCIT.^19^,^20^ Intraoperative hemorrhage remains a critical concern, particularly with vascular lesions, and meticulous hemostatic strategies—including bipolar coagulation and copious irrigation—are essential to safe resection.^19^,^21^ In select cases, neuro-navigation has further improved gross total resection (GTR) rates and minimized injury to deep venous structures.^19^

In our cohort, GTR was achieved in only 9.4%(3) of cases, reflecting the surgical complexity and anatomical constraints of PRT. Extent of resection often depends on tumor type, location, and operative approach. For instance, Tomita et al. reported GTR in 55 of 92 patients using a posterior interhemispheric OT approach, particularly in non-germinomatous tumors such as teratomas and astrocytomas, with favorable postoperative outcomes.^19^ In contrast, endoscopic SCIT approaches have demonstrated promising results in small series, including a 100% GTR rate in Hua et al.’s 4-patient study and 64.7% in Cai et al.’s larger microsurgical cohort.^20^,^22^ While GTR is generally associated with reduced recurrence in tumors like PPTID and Papillary tumor of the pineal region (PTPR), it may not significantly affect survival in germinomas or low-grade gliomas.^7^,^14^,^17^ The relatively low GTR rate in our study likely reflects a cautious surgical philosophy, prioritizing functional preservation over radical resection in eloquent midline structures, especially considering the relatively young age demographic of our patients.

Postoperative outcomes in patients with PRT are highly variable and depend on tumor pathology, extent of resection, and surgical approach. The most commonly diagnosed tumors in our cohort were germinomas and pineocytomas at 21.9%(7) each, followed by pineoblastoma 12.5%(4) and meningiomas 9.4%(3). In our cohort, 93.7%(30) of patients retained full motor strength postoperatively, with only two experiencing mild unilateral weakness. No cases of surgical site infection or new neurological deficits were reported, and the average post-operative KPS remained high at 91.5 ± 15.0, reflecting favorable short-term functional outcomes. Although long-term MRI follow-up was beyond the scope of this study, existing literature highlights a high recurrence rate in gliomas, PPTID, and particularly PTPR–where recurrence may reach up to 80% despite GTR.^7^,^17^ postoperative complications such as visual deficits, hemiparesis, and memory issues have been reported but are often transient and rarely life-threatening.^15^,^19^ Hormone replacement therapy is seldom necessary unless hypothalamic involvement is present, which remains uncommon across most series. Hospital stays typically range between 7-14 days depending on surgical complexity and complication rate.^18^

Functional outcomes following surgical intervention for PRT are closely linked to both the extent of resection and tumor histology. In our cohort, the high postoperative average KPS of 91.5 reflects excellent immediate functional recovery, aligning with literature that identifies KPS >60 as a significant predictor of long-term survival.^7^ Neurological recovery was near-complete in the vast majority of patients, with only two experiencing minor residual weakness—outcomes comparable to those seen in series employing navigation–guided and endoscopic techniques.^20^ Tumor-specific responses to adjuvant therapy further shape prognosis: germinomas typically respond very favorably to radiotherapy alone, while more aggressive pathologies such as PPTID,PTPR, and particularly pineoblastomas often require combined surgical resection and adjuvant radiotherapy.^14^,^18^ Chemotherapy remains limited in efficacy for most pineal parenchymal tumors and is generally reserved for refractory or metastatic disease. These findings underscore the importance of individualized, pathology-driven treatment plans in optimizing both survival and quality of life.

### Limitations:

It included a small sample size and retrospective data collection from patients’ records, which have multiple missing information, and this introduces information and selection biases. This study did not have any details of the surgical procedure due to short operative notes in the patient’s records, and no data on adjuvant therapies could be included in the study due to the provision of these outside our facility. Future studies with larger, more diverse samples are needed to apply the results to the general population and to further confirm the results.

## CONCLUSION

This study reinforces the clinical and surgical complexity of managing pineal region tumors, which present with symptoms of increased intracranial pressure. While gross total resection remains limited by anatomical constraints, favorable short-term neurological outcomes and high functional status are achievable through individualized, pathology-informed strategies. Continued integration of minimally invasive techniques, neuronavigation, and multidisciplinary care is essential for optimizing outcomes in this diverse tumor group.

### Clinical Recommendations:

Given the variability in tumor pathology and surgical accessibility, early recognition of obstructive hydrocephalus and prompt CSF diversion should be prioritized. Germinomas may be managed effectively with radiotherapy, while higher-grade lesions such as PPTID, PTPR, and pineoblastomas benefit from maximal safe resection followed by adjuvant therapy. Long-term surveillance with MRI remains critical due to recurrence risks in selective histology.

### Author`s Contribution:

**HMQ:** Concept and design of the work, data analysis and critical review of manuscript.

**AK, ZRN, ZMK, USK and RS:** Data acquisition and analysis, critically reviewed manuscript.

**RB and TS:** Data interpretation and drafted the manuscript.

**AM, SSHS and AB:** Concept and design of the study, critically reviewed the manuscript and supervision.

All authors have approved the final version to be published and agreement to be accountable for all aspects of the work in ensuring that questions related to the accuracy or integrity of any part of the work are appropriately investigated and resolved.
